# Sequestration of drugs in the circuit may lead to therapeutic failure during extracorporeal membrane oxygenation

**DOI:** 10.1186/cc11679

**Published:** 2012-10-15

**Authors:** Kiran Shekar, Jason A Roberts, Charles I Mcdonald, Stephanie Fisquet, Adrian G Barnett, Daniel V Mullany, Sussan Ghassabian, Steven C Wallis, Yoke L Fung, Maree T Smith, John F Fraser

**Affiliations:** 1 Critical Care Research Group, Adult Intensive Care Services, The Prince Charles Hospital & The University of Queensland, Rode Road, Chermiside, Queensland, Australia, 4032; 2 Burns Trauma and Critical Care Research Centre, The University of Queensland, Cnr Butterfield St and Bowen Bridge Rd, Herston, Queensland, Australia, 4029; 3 Institute of Health and Biomedical Innovation, School of Public Health & Social Work, Queensland University of Technology, Cnr Musk and Victoria Park Rd, Kelvin Grove, Queensland, Australia, 4059; 4 Centre for Integrated Preclinical Drug Development, Cnr Butterfield St and Bowen Bridge Rd Herston, The University of Queensland, Queensland, Australia, 4029

## Abstract

**Introduction:**

Extracorporeal membrane oxygenation (ECMO) is a supportive therapy, with its success dependent on effective drug therapy that reverses the pathology and/or normalizes physiology. However, the circuit that sustains life can also sequester life-saving drugs, thereby compromising the role of ECMO as a temporary support device. This *ex vivo *study was designed to determine the degree of sequestration of commonly used antibiotics, sedatives and analgesics in ECMO circuits.

**Methods:**

Four identical ECMO circuits were set up as per the standard protocol for adult patients on ECMO. The circuits were primed with crystalloid and albumin, followed by fresh human whole blood, and were maintained at a physiological pH and temperature for 24 hours. After baseline sampling, fentanyl, morphine, midazolam, meropenem and vancomycin were injected into the circuit at therapeutic concentrations. Equivalent doses of these drugs were also injected into four polyvinylchloride jars containing fresh human whole blood for drug stability testing. Serial blood samples were collected from the ECMO circuits and the controls over 24 hours and the concentrations of the study drugs were quantified using validated assays.

**Results:**

Four hundred samples were analyzed. All study drugs, except meropenem, were chemically stable. The average drug recoveries from the ECMO circuits and the controls at 24 hours relative to baseline, respectively, were fentanyl 3% and 82%, morphine 103% and 97%, midazolam 13% and 100%, meropenem 20% and 42%, vancomycin 90% and 99%. There was a significant loss of fentanyl (p = 0.0005), midazolam (p = 0.01) and meropenem (p = 0.006) in the ECMO circuit at 24 hours. There was no significant circuit loss of vancomycin at 24 hours (p = 0.26).

**Conclusions:**

Sequestration of drugs in the circuit has implications on both the choice and dosing of some drugs prescribed during ECMO. Sequestration of lipophilic drugs such as fentanyl and midazolam appears significant and may in part explain the increased dosing requirements of these drugs during ECMO. Meropenem sequestration is also problematic and these data support a more frequent administration during ECMO.

## Introduction

Extracorporeal membrane oxygenation (ECMO) is increasingly being used in adult patients with cardiac or respiratory failure refractory to conventional therapy or with both. ECMO can be an effective bridge to recovery, clinical decision-making, long-term mechanical cardiac support, and, less commonly, heart/lung transplantation [1]. Patients on ECMO receive multiple drugs that include sedatives and analgesics, antibiotics, anticoagulants, and vasoactive agents. The success of ECMO may rely on the successful use of these therapies. Although sedatives and vasoactive agents can be titrated to effect clinically, there are no reliable clinical markers to guide antibiotic therapy in critically ill patients. Antibiotics are commonly prescribed in patients on ECMO, and suboptimal therapy may result in therapeutic failure [2-5], adversely affecting patient outcomes. Despite the available endpoints for titration of sedation and analgesia in the intensive care unit [6] and efforts to minimize sedative drug use in this group [7], studies have reported escalating sedative doses over time in patients on ECMO [8-10].

There are limited data to guide drug therapy in adult patients receiving ECMO. Data from neonatal circuit experiments reveal significant sequestration of drugs in the ECMO circuit [11,12], and the extent of loss depends upon their physicochemical properties, type and age of the circuit, and the pumps used [10,13]. Pharmacokinetic (PK) studies in neonates [11,12] have consistently demonstrated increased volume of distribution (Vd) and decreased drug clearance (CL) during ECMO. Sequestration of drugs in the circuit appears to add to the increased Vd along with other factors related to critical illness, such as third spacing [11,14]. These studies in neonates highlight the important issue of altered PK during ECMO, but further extrapolation of the neonatal data to adult intensive care practice may not be relevant given the developmental and physiological differences between the two populations [15]. Systematic research in this area by using contemporary circuitry is required to develop evidence-based dosing guidelines for antibiotic therapy in adult patients receiving ECMO. The aim of this study was to describe the disposition of the analgesics fentanyl and morphine, the sedative agent midazolam, and the antibiotics meropenem and vancomycin in an *ex vivo *ECMO circuit model.

## Materials and methods

Ethics approval was obtained from the local Human Research Ethics Committee (HREC/12/QPCH/90).

### Extracorporeal membrane oxygenation circuits

The methods for the development of our *ex vivo *model of ECMO have been published previously [16]. Four permanent life support (PLS) ECMO circuits (Maquet Cardiopulmonary AG, Rastatt, Germany) were used. Each circuit consisted of Bioline tubing™, a PLS Quadrox D oxygenator, and RotaFlow pump head. A reservoir bladder (R-38; Medtronic Pty Ltd, Minneapolis, MN, USA) was added to allow fluid sampling from the closed circuit. The circuits were primed with 900 mL of Plasmalyte P-148 (Baxter Healthcare, Toongabbie, New South Wales, Australia) and then exchanged for 500 mL of Albumex 4 (Human Albumin 40 g/L; CSL Limited, Broadmeadows, Victoria, Australia). Porcine mucous heparin (Pfizer Australia, West Ryde, New South Wales, Australia) was added to the circuits (5,000 U). Fresh whole human blood (less than 5 days old, mean volume of 420 ± 52 mL, provided by Australian Red Cross Blood Service, Melbourne, Victoria, Australia) was used for the final prime, and the circuits were pressurized to obtain post-oxygenator pressures of 230 to 250 mm Hg.

The final volume of the pressurized circuit was 668 ± 1.7 mL, and the mean hemoglobin value was 64 ± 13 g/L. The mean total protein and albumin concentration in the circuit were 33 ± 2.5 g/L and 25 ± 0.9 g/L, respectively. Activated clotting time was maintained between 220 and 250 seconds. A centrifugal pump (Jostra RotaFlow; Maquet Cardiopulmonary AG) was used to maintain a circuit flow rate of 4 to 5 L/minute. Oxygen tension in the circuit blood was maintained between 150 and 200 mm Hg. Circuit temperature was maintained at 37°C. Carbon dioxide gas or sodium bicarbonate solution was added to the circuit to maintain the pH of the circulating blood in the range of 7.25 to 7.55. Fentanyl (20 μg), morphine (100 μg), midazolam (100 μg), meropenem (10 mg), vancomycin (40 mg), propofol (1 mg), dexmedetomidine (5 μg), thiopentone (20 mg), ceftriaxone (50 mg), linezolid (10 mg), ciprofloxacin (5 mg), fluconazole (10 mg), and caspofungin (5 mg) were injected post-oxygenator as a single bolus. The drugs with known incompatibilities to study drugs (for example, gentamicin and ticarcillin/clavulunate) were excluded. These bolus doses were selected to produce concentrations similar to clinical concentrations. Larger doses were used for the drugs that exhibit high protein binding.

### Controls

Four polyvinylchloride jars with tight caps were filled with 50 mL of fresh human whole blood. Unfractionated heparin (500 U) was added to the jars for anticoagulation. Fentanyl (1.5 μg), morphine (7.5 μg), midazolam (7.5 μg), meropenem (0.75 mg), vancomycin (3 mg), propofol (75 μg), dexmedetomidine (0.375 μg), thiopentone (1.5 mg), ceftriaxone (3.75 mg), linezolid (0.75 mg), ciprofloxacin (0.375 mg), fluconazole (0.75 mg), and caspofungin (0.375 mg) were added to the control jars after collection of baseline blood samples. These amounts were chosen in order to produce study drug concentrations that were similar to those achieved in the ECMO circuit. The jars were then placed in an incubator at 37°C and rocked continuously to ensure even distribution of the drugs.

### Blood sample collection

Post-oxygenator blood was collected into lithium heparin tubes (5 mL) at baseline and at 2, 5, 15, 30, 60, 120, and 360 minutes and at 12 and 24 hours after addition of the drugs to the circuit. Blood samples (5 mL) were also obtained from the control jars at time intervals identical to that of the circuit. All blood samples were stored on ice and centrifuged (10 minutes at 3,000*g*), and the plasma was separated and stored in clean pre-labeled polypropylene cryogenic vials and stored at -80°C until analysis.

### Measurement of drugs in plasma samples

An on-line solid-phase extraction (SPE) Symbiosis Pharma system (Spark Holland, Emmen, The Netherlands) was used to extract the analytes of interest (fentanyl, morphine, and midazolam) and two internal standards (morphine-d3 and 1-hydroxymidazolam-d5) from plasma samples simultaneously [17]. Mass spectrometry in ESI (electrospray ionization) mode (API 5500; AB Sciex, Framingham, MA, USA) triple quadropole system was used as the detector. Liquid chromatography and extraction method were created by Symbiosis Pro for Analyst (version 2.1.0.0) and submitted to the MS controlling software (Analyst 1.5.1). Meropenem and vancomycin concentrations in the collected plasma samples were determined by separate validated chromatographic assay methods. Meropenem and the internal standard (cefotaxime) were detected by ultraviolet absorbance at 304 nm. Vancomycin analysis was by liquid chromatography-tandem mass spectrometry on an Applied Biosystems API2000 (Applied Biosystems, Foster City, CA, USA) with Shimadzu autosampler (Shimadzu Corporation, Kyoto, Japan). Vancomycin and the internal standard (teicoplanin) were detected by positive-mode MRM (multiple reaction monitoring). All samples were assayed alongside calibration standards and quality control samples and met the acceptance criteria.

### Statistical analysis

Linear mixed effects modeling was used to examine the change in concentration over time. This model accounts for the repeated responses from the same circuit by using a random intercept. The mixed effects model was fitted by using the R statistical software [18] version 2.13.2 and the 'lme4' library. The concentration-versus-time curves (mean ± standard error of the mean) were plotted by using GraphPad Prism version 5.03 (GraphPad Software, Inc., La Jolla, CA, USA).

## Results

**Figure 1:**
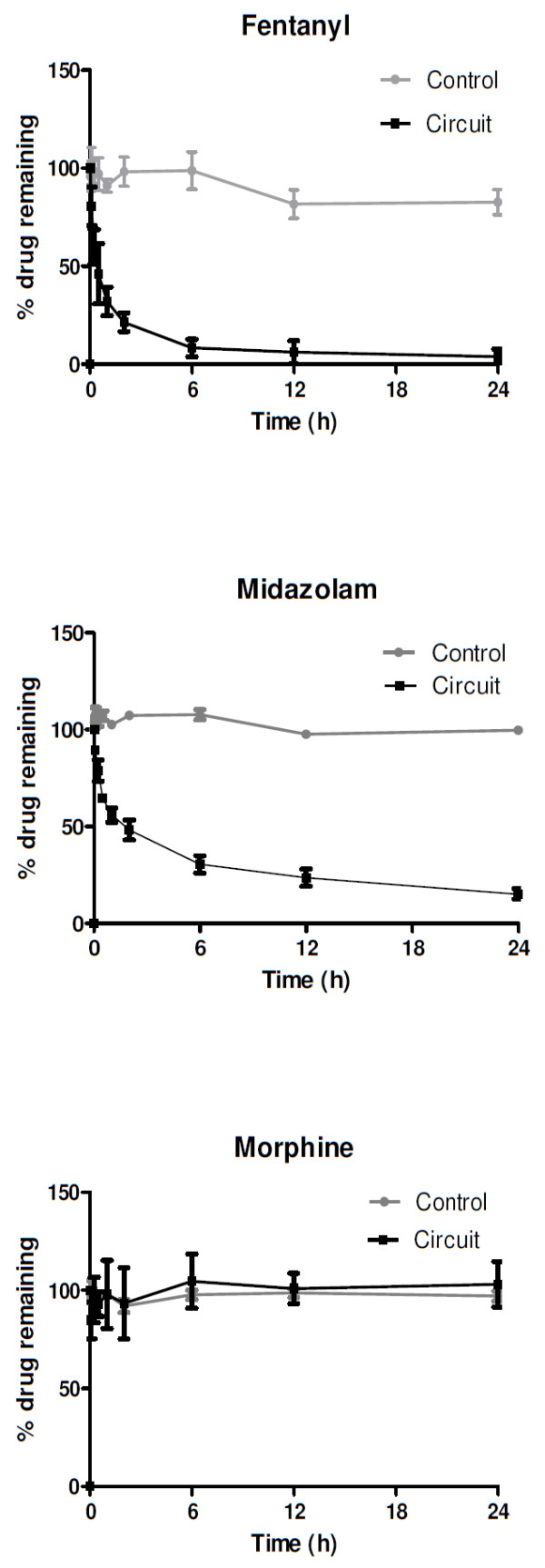
**Percentage of drug remaining in extracorporeal membrane oxygenation circuits and the controls plotted against time**. Lipohilic drugs such as fentanyl and midazolam were significantly sequestered in the circuit despite being stable in the controls. Morphine was relatively stable in both controls and the circuits.

**Figure 2:**
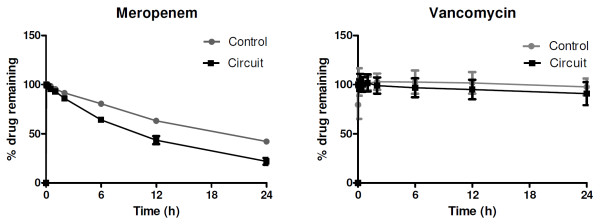
**Percentage of meropenem and vancomycin remaining in extracorporeal membrane oxygenation circuits and the controls plotted against time**. Meropenem was unstable in the controls; however, the extracorporeal membrane oxygenation circuit losses significantly exceeded the levels of degradation seen in the controls at 6 hours. Vancomycin was stable in both controls and the circuits.

The *ex vivo *circuits were maintained under physiological conditions for 24 hours with no complications during the run. Four hundred samples (80 per drug) were analyzed. The changes in drug concentrations in the *ex vivo *model are summarized in Figures 1 and 2, and the actual drug concentrations determined in plasma samples from the control jars and the ECMO circuits are presented in Table 1. In this paper, only the data for fentanyl, morphine, midazolam, meropenem, and vancomycin are presented. Validated assays are being developed for the remaining study drugs, and the results will be made available in due course.

**Table 1:** Measured study drug concentrations in the control jars and the extracorporeal membrane oxygenation circuits at 5 minutes and at 1, 6, 12, and 24 hours

Drug, units of concentration	5 minutes		1 hour		6 hours		12 hours		24 hours	
	**C**	**E**	**C**	**E**	**C**	**E**	**C**	**E**	**C**	**E**

Fentanyl, ng/mL										

Median	27	1	25	0.4	26	0.1	22	0.1	22	0

Range	26-29	1-1	24-25	0.2-0.5	26-28	0-0.2	20-24	0-0.2	20-24	0-0.1

Morphine, ng/mL										

Median	136	7	139	8	137	8	139	8	135	8

Range	134-140	6-7	132-141	7-9	134-140	8-9	135-143	8-8	134-141	7-9

Midazolam, ng/mL										

Median	134	175	131	117	138	59	124	42	128	31

Range	126-155	144-356	124-137	109-261	127-147	54-124	117-132	41- 96	120-131	30-61

Vancomycin, μg/mL										

Median	71	73	70	73	72	71	72	71	69	67

Range	66-80	67-83	69-76	70-86	67-76	69-74	68-74	68-71	67-70	64-70

Meropenem, μg/mL										

Median	18	15	17	14	14	10	11	6	7	3

Range	17-18	11-16	16-18	10-15	13-15	7-11	11-11	4-8	7-8	2-5

C, control jars; E, extracorporeal membrane oxygenation circuits.

Testing confirmed that all baseline plasma samples were free of study drugs. There were no statistically significant differences in drug recoveries between the four circuits. The mean drug recoveries from the circuits and the control jars at 24 hours relative to baseline were, respectively, fentanyl 3% and 82%, morphine 103% and 97%, midazolam 13% and 100%, meropenem 20% and 42%, and vancomycin 90% and 99%. Up to 70% of fentanyl and 50% of midazolam were lost in the circuit within the first hour of ECMO run. Fentanyl levels were undetectable in the circuit by 24 hours. This may be related to the lipophilicty of these drugs. Morphine, which is less lipophilic than fentanyl, was stable in both the circuit and the controls. Antibiotics were less significantly affected. The hydrophilic and minimally protein-bound drug meropenem was stable in the circuit and the controls in the first 120 minutes, and 62% of the drug was recovered from the circuit at 6 hours. This was statistically significant (*P *= 0.01) even after accounting for spontaneous degradation (21%). There was no significant loss of the moderately protein-bound hydrophilic drug vancomycin in the circuit at 12 hours (*P *= 0.41) or 24 hours (*P *= 0.26).

## Discussion

This is the first systematic investigation of drug disposition in the adult ECMO circuitry. The findings highlight the role of the circuit in altering the PK of sedative, analgesic, and antibiotic drugs during ECMO and clearly show that there is considerable between-drug variability in the degree of drug sequestration. Drugs that are unstable at physiological temperature (meropenem) and lipophilic drugs (fentanyl and midazolam) were more significantly affected. These findings may have significant implications for both the choice and the dosing of a particular drug prescribed during ECMO. Given the ongoing exteriorization of blood onto the circuit during ECMO, *in vivo *instability of drugs may also play a significant role in apparent PK during ECMO. By excluding the patient factors, this *ex vivo *model provides evidence that the adult ECMO circuit is not simply a benign conduit for blood but actively modulates drug PK.

The circuit factors were identical for all drugs. In this context, it is difficult to determine which of the drug factors contributed to the significant disparity in the degree of drug sequestration in the circuit and *ex vivo *stability. Differences in molecular size and lipophilicity and the differences in protein binding may all have contributed to the findings. This is important as a blanket increase in doses of all antibiotic drugs to avoid under-dosing without identifying the drugs that are most sequestered by the ECMO circuit may potentially result in drug toxicity. Similarly, drug sequestration in the circuit may also explain the increasing sedation requirements seen in patients on ECMO [8,9]. Using sedative and analgesic agents that are highly sequestered in the circuit may necessitate the use of very high doses of these drugs to achieve the desired pharmacological effect and may add to the associated morbidity [6]. This calls for further research in this area to improve drug prescription during ECMO.

Given that meropenem and vancomycin both rely on time-dependent bacterial killing, the data presented here on altered antibiotic concentrations may be clinically relevant and require evaluation in a clinical PK study. Meropenem is degraded and sequestered significantly in the circuit beyond 4 to 6 hours. Hence, a more frequent dosing or use of higher doses may be required to maximize the time above minimum inhibitory concentration of the pathogen [19] as demonstrated in a recent clinical study [20]. Furthermore, administration of meropenem by infusion is questionable given the instability issues at room temperatures [21]. The utility of more stable carbapenem antibiotics such as doripenem may have to be explored in future studies. Clinically, there are no data on meropenem PK in patients receiving ECMO. Neonatal studies have uniformly shown an increase in Vd for vancomycin and a lower CL and consequently a longer vancomycin half-life [22,23]. Similarly, PK studies in neonates have shown increased Vd and decreased CL for morphine, midazolam, and their metabolites during ECMO [24,25]. The extent to which these PK alterations during ECMO are related to sequestration of these drugs in the circuit is currently unclear.

Studies in the neonatal ECMO circuits have demonstrated significant sequestration of sedative and antibiotic drugs in the circuit. Also, there is drug sequestration variability based on the different circuits, oxygenators, and pumps used [13]. A recent *in vitro *study [13] reported meropenem and vancomycin recovery of 89% and 67% at 180 minutes in neonatal circuits that used a centrifugal pump and polypropylene hollow-fiber membrane oxygenators. Whereas the meropenem recovery at 180 minutes is comparable to our results, vancomycin recovery was much lower in the neonatal circuits. In contrast, the fentanyl and midazolam circuit losses seen in this study are consistent with the results of the neonatal circuit studies [13,26-28]. Morphine appears to be relatively stable in both neonatal and adult ECMO circuits and may be the preferred analgesic during ECMO. Future clinical studies should compare the efficacy of different classes of drugs to rationalize sedation and analgesia during ECMO.

Studies that compare drug losses in clinically used versus new neonatal circuits have demonstrated significant variability in drug sequestration between the used and new circuits [10,13,28]. Consequently, it is still unclear whether there is saturation of the drug-binding sites in the ECMO circuit over time. Similarly, the effect of priming with various solutions on drug sequestration is not well characterized in the available literature. Drug sequestration in blood-primed circuits has been shown to be much lower than that in crystalloid-primed circuits [27]. It is possible that some of the blood components may compete with drugs for circuit-binding sites. Even though ECMO circuits are not routinely primed with blood prior to their use in adult patients, the circuits get primed with the patient's own blood once ECMO is commenced.

In our study, we tried to replicate the clinical situation *ex vivo *which allowed us to study the interactions between the drug and the device in the absence of disease-related factors which independently can significantly alter PK [29,30]. Repeat dose experiments are required in long-term model systems to estimate the degree of circuit saturation with time. The concurrent presence of several other physically compatible study drugs in the circuit and control jars mimicked the clinical scenario in which patients receive these drugs concurrently, but the drugs may have had an impact on competitive binding to blood proteins or the circuit components. The presence of a reservoir bladder may have influenced the circuit drug losses. Similarly, quantification of drug lost in control jars because of binding of drugs to the polypropylene container was not feasible. However, the results confirm the findings of neonatal ECMO circuit studies.

## Conclusions

This *ex vivo *study highlights the role of the ECMO circuit in altering PK during ECMO. These alterations are more pronounced for lipophilic drugs and may result in therapeutic failure. Morphine may be a useful alternative to fentanyl in a patient with escalating sedative and analgesic drug requirements. Less lipophilic sedative agents may have to be considered in patients receiving unusually high doses of midazolam. Given the instability issues and circuit sequestration, meropenem may have to be dosed more frequently or in higher doses pending future clinical PK studies. Vancomycin is less significantly affected, and therapeutic drug monitoring as currently practiced can guide optimal treatment. PK studies in adult patients on ECMO are indicated for future research in order to generate the data to guide antibiotic, sedative, and analgesic therapy during ECMO.

## Key messages

• Lipophilic drugs appear to be more significantly sequestered in the ECMO circuit, although further study with different lipophilic drugs is required to confirm this observation.

• Fentanyl and midazolam are more significantly sequestered than morphine.

• Meropenem may have to be administered more frequently during ECMO.

• Physical instability of meropenem may affect its delivery by a continuous infusion.

• Sequestration of drugs in the circuit may have implications on both the choice and dosing of a particular drug prescribed during ECMO.

## Abbreviations

CL: clearance; ECMO: extracorporeal membrane oxygenation; PK: pharmacokinetics; PLS: permanent life support; Vd: volume of distribution.

## Competing interests

The authors declare that they have no competing interests.

## Authors' contributions

KS designed and coordinated the study, collected and analyzed data, and developed the manuscript for publication. CIM provided technical assistance in setting up the circuits. SF helped with procuring study drugs and dispensing them. AGB helped with statistical analysis. SG assayed study drugs midazolam, morphine, and fentanyl. SCW assisted with the control experiments and antibiotic drug assays. MTS and JAR helped with study design, data analysis, and manuscript preparation. DVM, YLF, and JFF assisted with study design, resources, and manuscript preparation. All authors read and approved the final manuscript.
